# Supplementary material for: Genetic Diversity in the Suppressyn Gene Sequence: From Polymorphisms to Loss-of-Function Mutations
Source: Biomolecules. 2025 Jul 21;15(7):1051. doi: 10.3390/biom15071051 (PMC12293451; doi:10.3390/biom15071051)
Supplement: Supplementary file 1 [file biomolecules-15-01051-s001.zip › biomolecules-3701529 Supplymentary.pdf]

SUPYN ID	c.47	c.79	c.385	c.394	c.427	c.449
Genome region	44,339,070	44,339,038	44,338,732	44,338,723	44,338,690	44,338,668
Genotype	C=0.0005<br>T=0.9995	G=0.0510<br>T=0.9490	G=0.0023<br>T=0.9977	C=0.0124<br>G=0.9876	C=0.0014<br>T=0.9986	A=0.0234<br>G=0.9766
SNP ID	rs141435072	rs62220807	rs148319804	rs116336164	rs188629514	rs77912643
Amino acid substitution	p.K16R	p.I27L	p.K129Q	p.Q132E	p.T143A	p.P150L

Supplementary Figure S1
Details of single nucleotide variants (SNVs) identified within the *suppressyn* coding sequence using the 1000 Genomes Project database.

a)

Sequence
Prediction: Signal Peptide (Sec/SPI)

Cleavage site between pos. 39 and 40. Probability 0.909469

Protein type	Other	Signal Peptide (Sec/SPI)	Lipoprotein signal peptide (Sec/SPII)	TAT signal peptide (Tat/SPI)	TAT Lipoprotein signal peptide (Tat/SPII)
**Likelihood**	0.0183	0.9809	0.0002	0.0002	0.0001

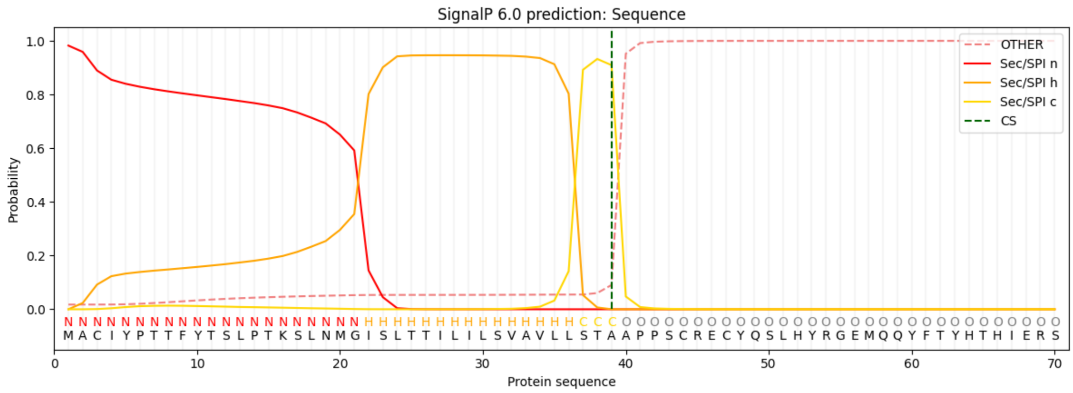

b)

```
Name:  1        Length:  160
MACIYPTTFYTSLPTKSLNMGISLTTILILSVAVLLSTAAPPSCRECYQSLHYRGEMQQYFTYHTHIERSCYGNLIEECV      80
ESGKSYYKVKNLGVCGSRNGAICPRGKQWLCFTKIGQWGVNTQVLEDIKREQIIAKAKASKPTTPPENRPRHFHSFIQKL     160
................................................................................      80
..............................................................G.................     160
```

Name	Residue	Number	Potential	Threshold	Assignment
1	Thr	0007	0.0846	0.6585	.
1	Thr	0008	0.0108	0.6325	.
1	Thr	0011	0.0966	0.6075	.
1	Ser	0012	0.0141	0.5925	.
1	Thr	0015	0.1169	0.5386	.
1	Ser	0017	0.2276	0.5306	.
1	Ser	0023	0.3285	0.7344	.
1	Thr	0025	0.0276	0.7833	.
1	Thr	0026	0.0195	0.7823	.
1	Ser	0031	0.3270	0.8113	.
1	Ser	0037	0.0249	0.6345	.
1	Thr	0038	0.0593	0.5935	.
1	Ser	0043	0.0417	0.4637	.
1	Ser	0050	0.0206	0.6225	.
1	Thr	0062	0.0115	0.6824	.
1	Thr	0065	0.0115	0.6605	.
1	Ser	0070	0.0199	0.6125	.
1	Ser	0082	0.0151	0.5076	.
1	Ser	0085	0.0958	0.4756	.
1	Ser	0097	0.0291	0.5586	.
1	Thr	0113	0.0275	0.6714	.
1	Thr	0122	0.0165	0.6684	.
1	Ser	0140	0.0170	0.3927	.
1	Thr	0143	0.3706	0.3618	G
1	Thr	0144	0.1426	0.3498	.
1	Ser	0155	0.0802	0.6625	.

Supplementary Figure S2

a) Predicted signal peptide sequence within the suppressyn protein, analyzed using SignalP-6.0 (https://services.healthtech.dtu.dk/services/SignalP-6.0/).
b) Predicted O-glycosylation sites identified using NetOGlyc-4.0 (https://services.healthtech.dtu.dk/services/NetOGlyc-4.0/).

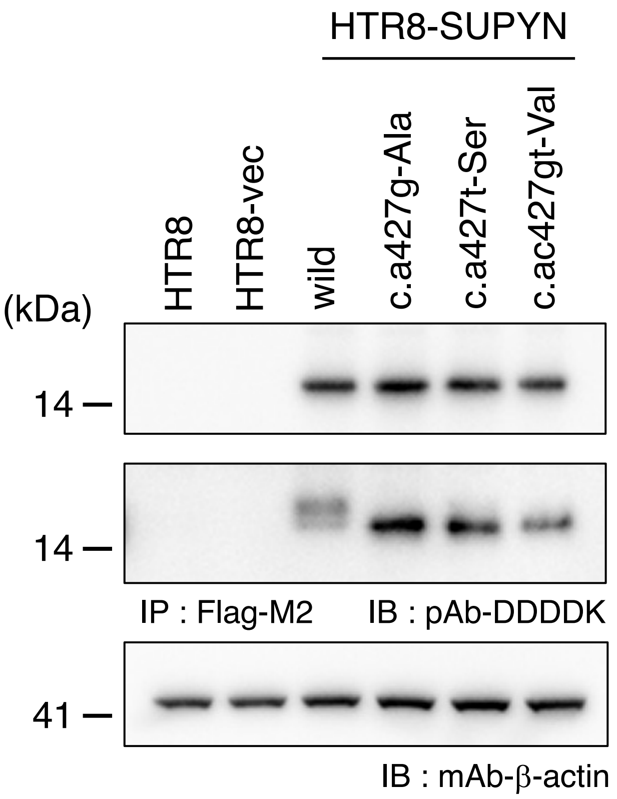

Supplementary Figure S3
Validation of O-glycosylation sites.
To evaluate O-glycosylation at predicted sites, a series of mutant constructs were generated. These included a threonine-to-serine substitution (Thr→Ser), which is predicted to partially retain O-glycosylation, and threonine-to-alanine (Thr→Ala) or threonine-to-valine (Thr→Val) substitutions, which are expected to result in complete loss of glycosylation. HTR8 cells were transiently transfected with these constructs, and after two days of culture, suppressyn protein in both the cell lysates and culture supernatants was analyzed by Western blotting, following the same protocol as in Figure 1. 'Cell' indicates the cell lysate, and 'sup.' indicates the culture supernatant. Primers for O-glycosylation mutagenesis are listed in Supplementary Table S1. Original full-length blots for SDS-PAGE analyses are provided in Supplementary Figure S11.

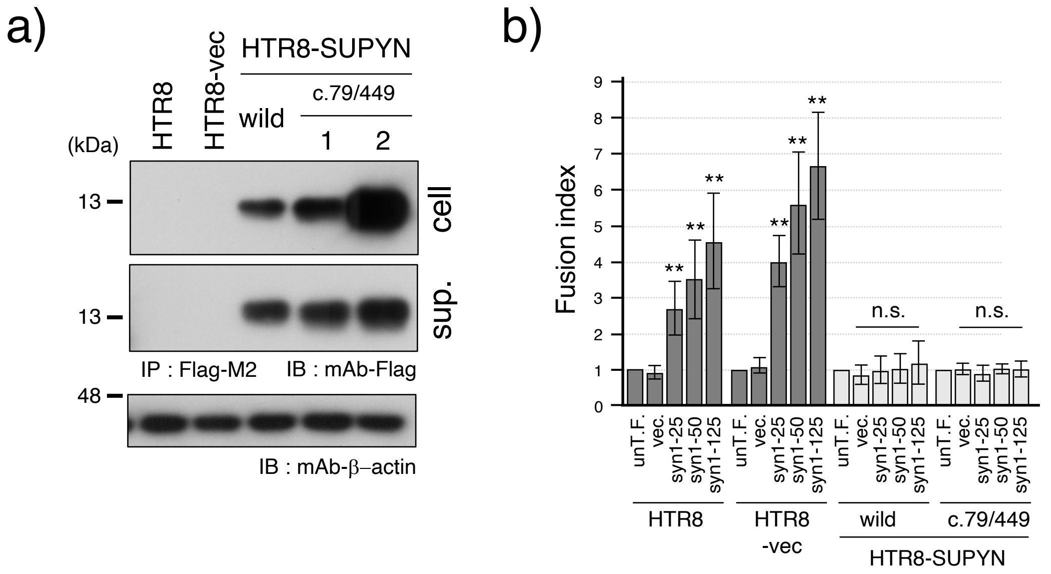

Supplementary Figure S4

Functional analysis of suppressyn harboring both c.79 and c.449 SNVs.

a) Western blot analysis of suppressyn proteins with c.79/449 mutations expressed in HTR8 cells. Proteins were detected in cell lysates and culture supernatants after immunoprecipitation using anti-Flag M2 agarose. 'Cell' indicates the cell lysate, and 'sup.' indicates the culture supernatant. b) Cell fusion assay using suppressyn-expressing cells. Syncytin-1 plasmid was transfected at 25, 50, and 125 ng, and fusion efficiency was quantified by FACS analysis. Data are normalized to un-transfected controls (un-T.F. = 1). Statistical significance was assessed by Mann–Whitney U test (*$p < 0.05$, **$p < 0.01$, n.s. = not significant). The abbreviation 'un-T.F.' refers to un-transfected cell. Error bars represent the standard deviation (SD). All experiments were performed in duplicate and repeated at least three times. The numbers 1 and 2 (c.79/449-1, c.79/449-2) shown in the figure indicate duplicates generated using two independent clones. Original full-length blots for SDS-PAGE analyses are provided in Supplementary Figure S12.

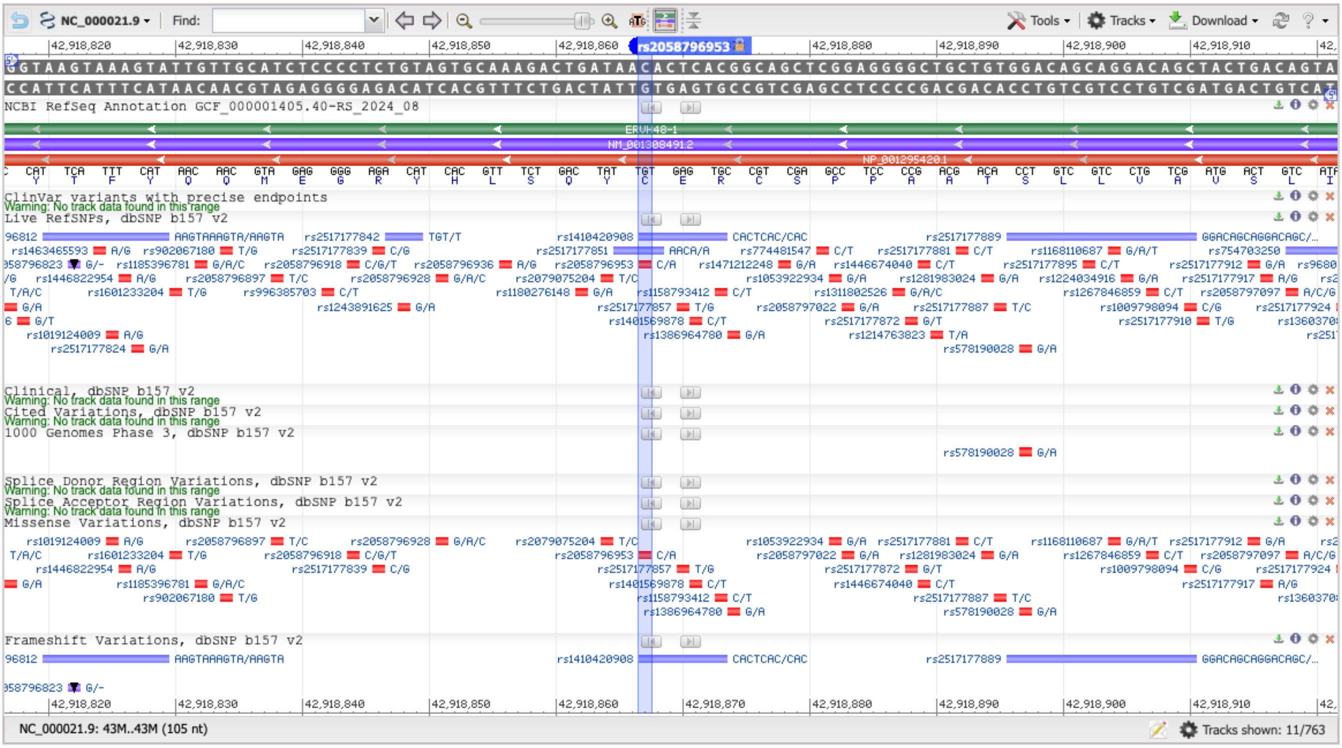

Supplementary Figure S5

A missense SNV (rs2058796953) involving the Cys#2 residue of *suppressyn* identified in the NCBI dbSNP database (https://www.ncbi.nlm.nih.gov/snp/).

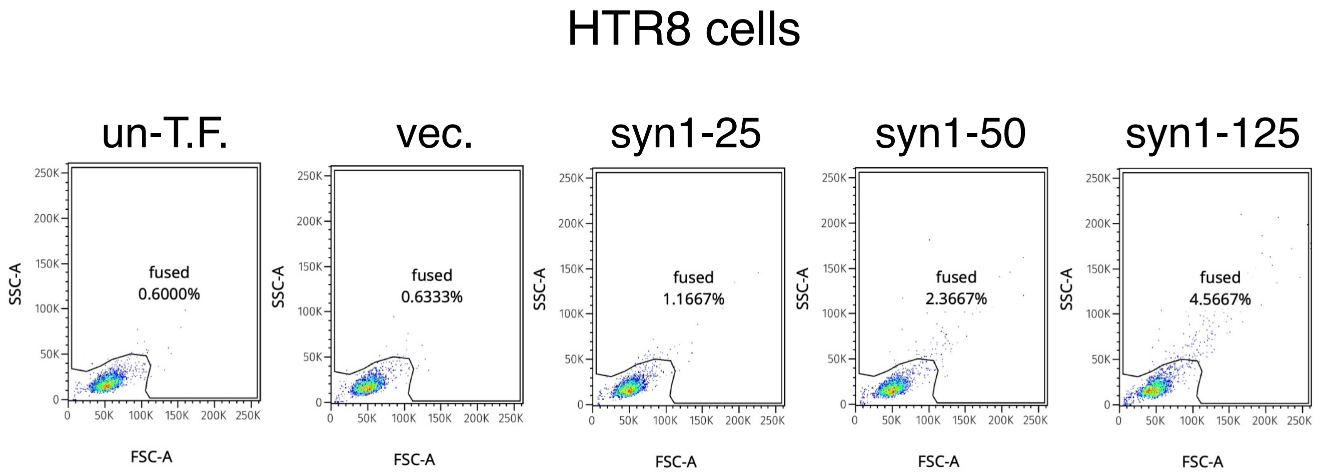

Supplementary Figure S6
Example of cell fusion rate detection by flow cytometry.

Prepared cells were analyzed using flow cytometry (FACSVerse; BD Biosciences, NJ, USA), and populations were separated based on standard forward and side scatter (FSC vs SSC) parameters, which reflect cell size and internal complexity. A baseline population was established using control un-transfected HTR8 cells (left panel : un-T.F.), serving as a reference for non-fused cells. Based on this reference, a gate was drawn to define a population of larger-sized cells corresponding to fused cells (indicated as "fused" in the figure). HTR8 cells transfected with an empty vector, as well as cells transfected with increasing amounts of syncytin-1 plasmid (25–125 ng), were processed similarly. The percentage of fused cells was quantified within the defined fused gate.

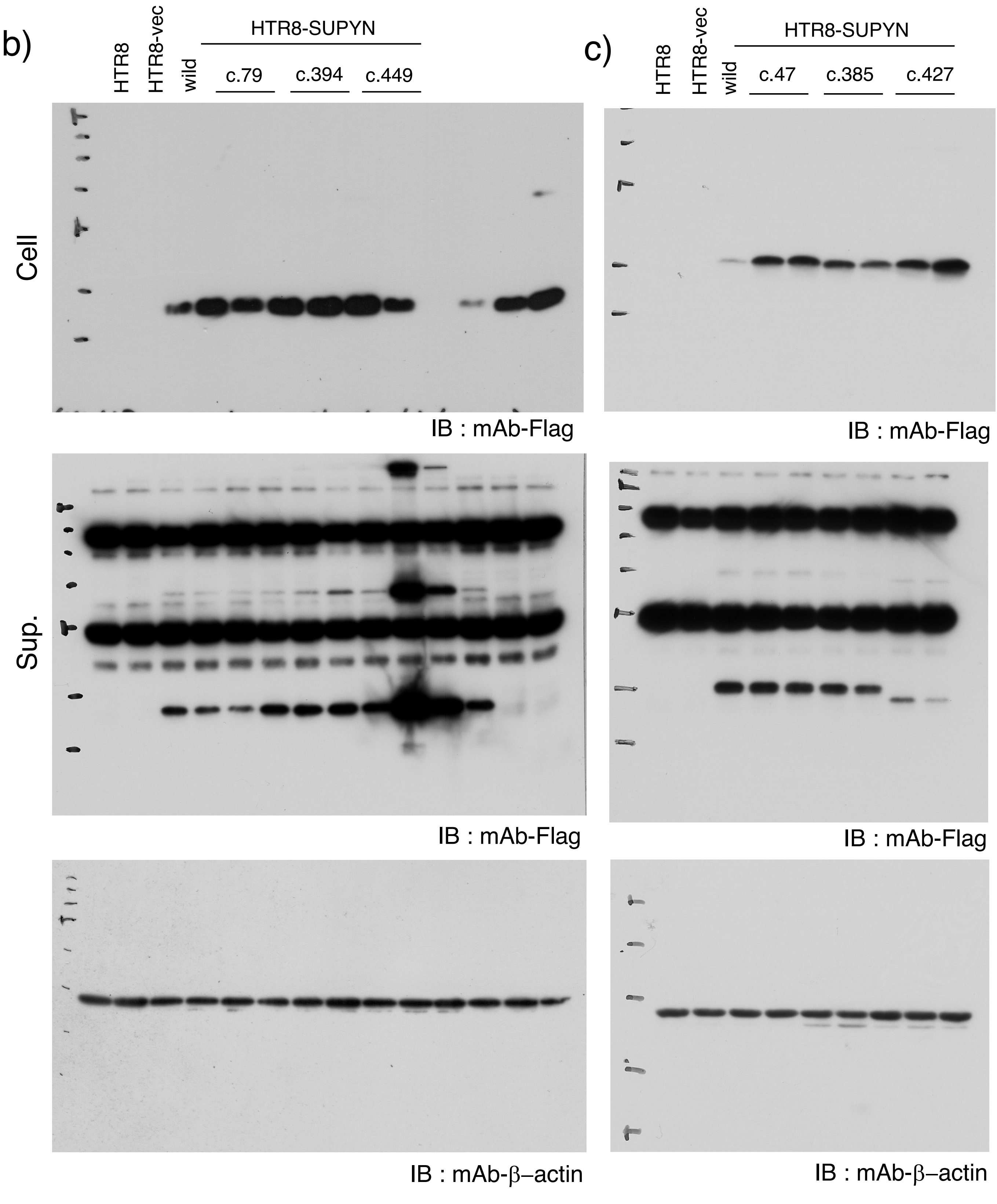

Supplementary Figure S7

Full immunoblot images corresponding to Figure 1b and 1c.

a)

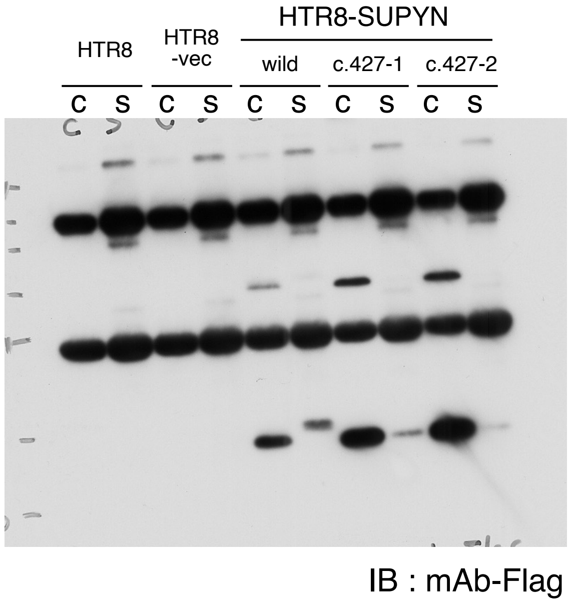

b)

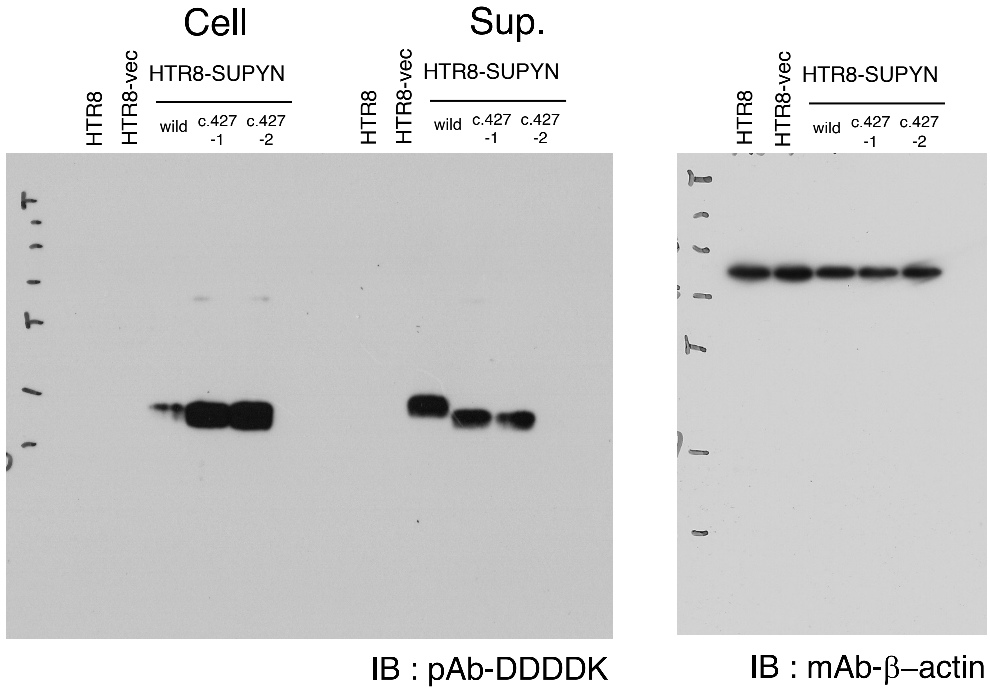

Supplementary Figure S8

Full immunoblot images corresponding to Figure 2a and 2b.

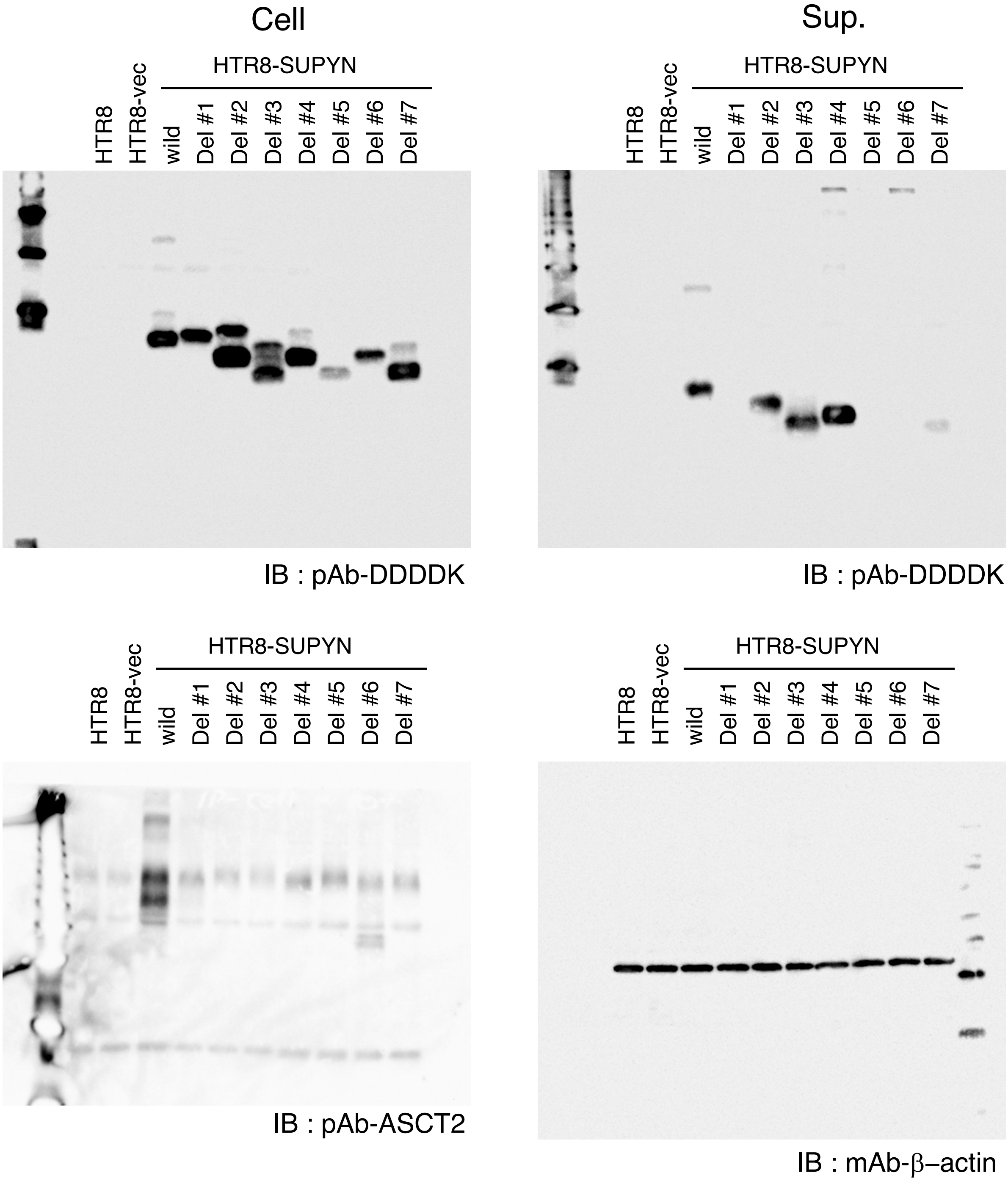

Supplementary Figure S9

Full immunoblot images corresponding to Figure 3b.

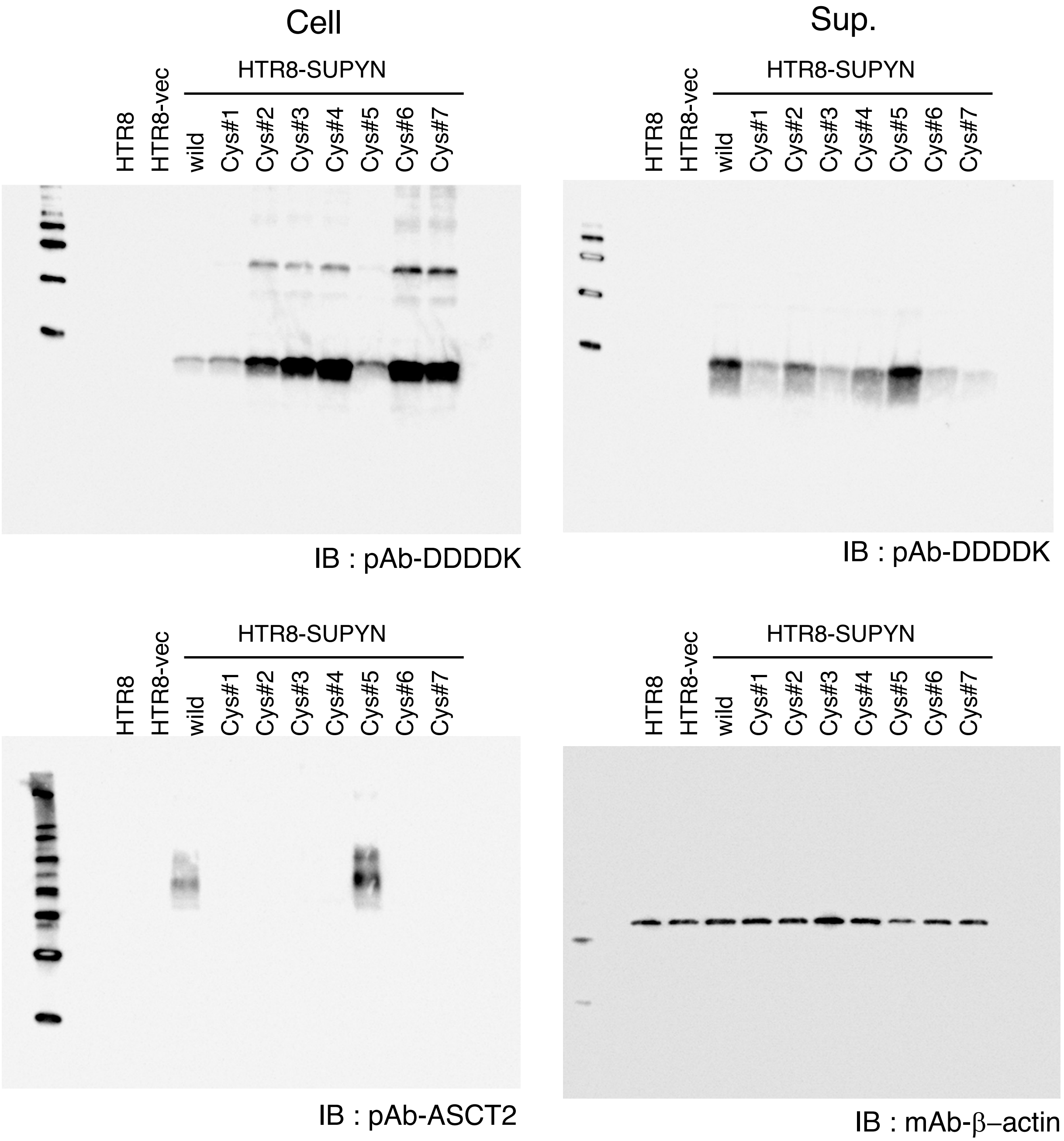

Supplementary Figure S10

Full immunoblot images corresponding to Figure 4b.

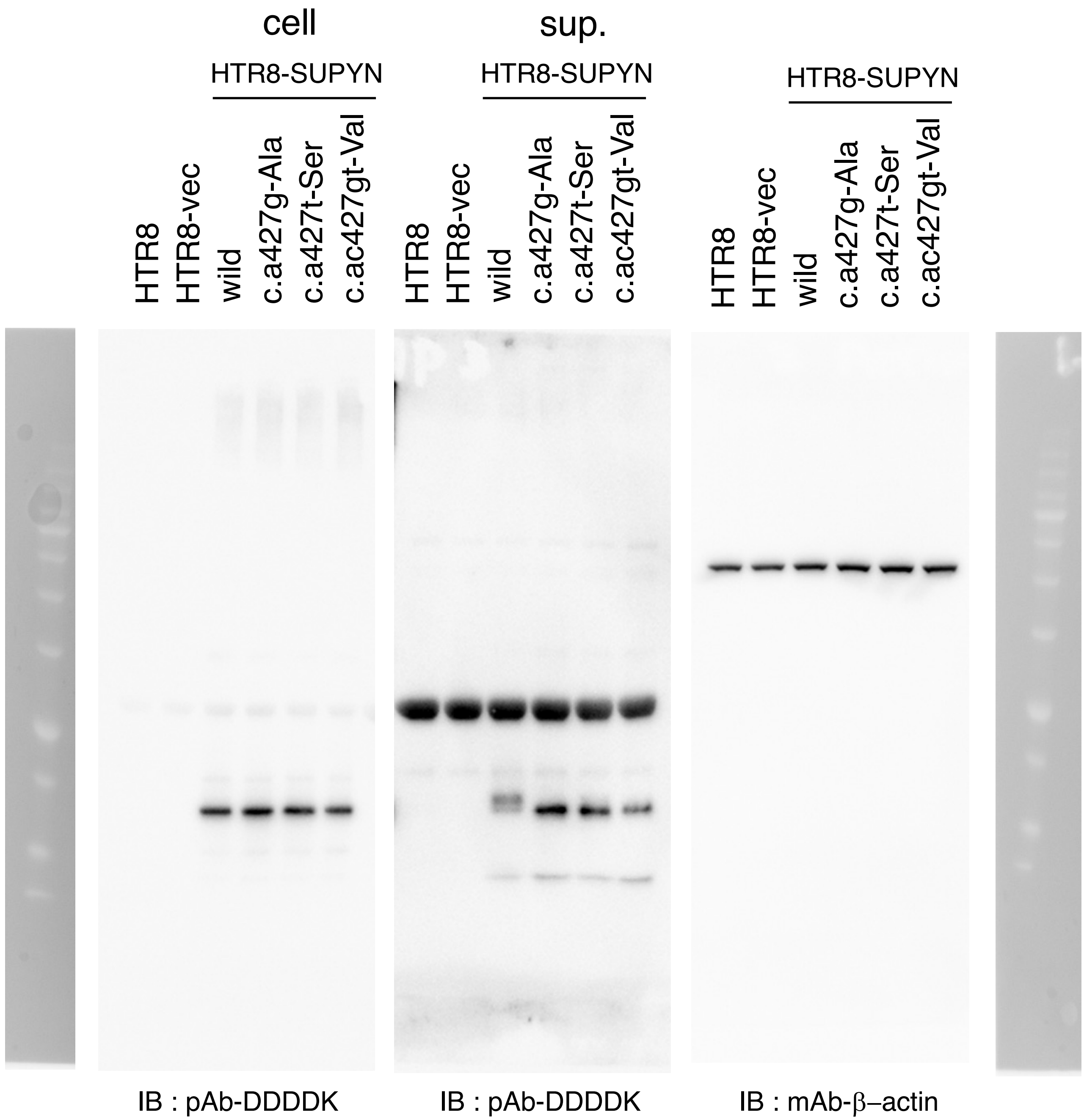

Supplementary Figure S11

Full immunoblot images corresponding to Supplementary Figure S3.

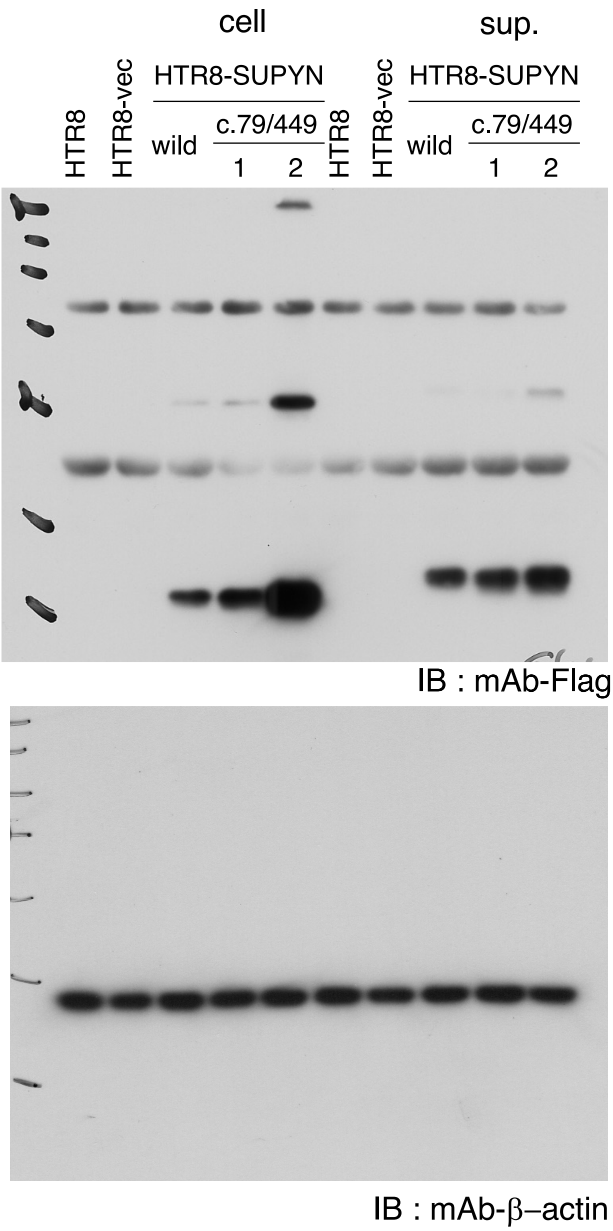

Supplementary Figure S12

Full immunoblot images corresponding to Supplementary Figure S4.

Cloning	Primer ID	Sequence (5' - 3')
SNP	SUPYN-a79c-S	caccacgCtcctaatactgtcagtag
	SUPYN-a79c-AS	attaggaGcgtggtgagggatattcc
	SUPYN-c394g-S	gagagaaGagattatagccaaagcca
	SUPYN-c394g-AS	ataatctCttctctctttatgtcctc
	SUPYN-c449t-S	aatcgccTgcggcatttccattcctt
	SUPYN-c449t-AS	atgccgcAggcgattttcagggggag
	SUPYN-a47g-S	ccaaccaGaagtcttaatatgggaata
	SUPYN-a47c-AS	aagacttCtggttggaagagaggtata
	SUPYN-a385c-S	ggacataCagagagaacagattatagc
	SUPYN-a385c-AS	tctctctGtatgtcctcaagcacctga
	SUPYN-a427g-S	aaaaccaGcaactccccctgaaaatcg
	SUPYN-a427g-AS	ggggagttgCtggttttgaggctttggct
Deletion	SUPYN-del7-39-S	ctacccagcccctccgagctgccgt
	SUPYN-del7-39-AS	ggaggggctgggtagatacaggccat
	SUPYN-del40-66-S	cacagcaatagaaagatcctgttat
	SUPYN-del40-66-AS	ctttctattgctgtggacagcaggac
	SUPYN-del118-144-S	tggacaacccccctgaaaatcgcccg
	SUPYN-del118-144-AS	tcaggggggttgtccaattttggtgaa
	SUPYN-del67-117-S	tactcattggggagtaaacactcag
	SUPYN-del67-117-AS	actccccaatgagtatggtaagtaaa
Cystein	SUPYN-Cys#1-S	ccgagctgGcgtgagtgttatcagtct
	SUPYN-Cys#1-AS	ctcacgCcagctcggaggggctgctgt
	SUPYN-Cys#2-S	cgtgagtgGtatcagtctttgcactac
	SUPYN-Cys#2-AS	ctgataCcactcacggcagctcggagg
	SUPYN-Cys#3-S	agatcctgGtatggaaacttaatcgag
	SUPYN-Cys#3-AS	tccataCcaggatctttctatatgagt
	SUPYN-Cys#4-S	gaggaatgGgttgaatcaggaaagagt
	SUPYN-Cys#4-AS	ttcaacCcattcctcgattaagtttcc
	SUPYN-Cys#5-S	ggagtatgGggcagtcgtaatggggct
	SUPYN-Cys#5-AS	actgccCcatactcctagattctttac
	SUPYN-Cys#6-S	gctatttgGcccagagggaagcagtgg
	SUPYN-Cys#6-AS	tctgggCcaaatagccccattacgact
	SUPYN-Cys#7-S	tggctttgGttcaccaaaattggacaa
	SUPYN-Cys#7-AS	ggtgaaCcaaagccactgcttccctct
O-Glycosylation	SUPYN-a427t Ser-S	aaaaccaTcaactccccctgaaaatcg
	SUPYN-a427t Ser-AS	ggggagttgAtggttttgaggctttggct
	SUPYN-ac427gt Val -S	aaaaccaGTaactccccctgaaaatcg
	SUPYN-ac427gt Val -AS	ggggagttACtggttttgaggctttggct

Supplementary Table S1

Primer sequences used for the construction of *suppressyn* mutants.
